# Transcriptome Analyses Revealed the Key Metabolic Genes and Transcription Factors Involved in Terpenoid Biosynthesis in Sacred Lotus

**DOI:** 10.3390/molecules27144599

**Published:** 2022-07-19

**Authors:** Lili Qin, Fei Du, Ningning Yang, Chen Zhang, Zhiwen Wang, Xingwen Zheng, Jiawei Tang, Liangbo Yang, Chen Dong

**Affiliations:** 1 College of Biological Engineering, Henan University of Technology, Zhengzhou 450001, China; qin19971204@163.com (L.Q.); 15737321533@163.com (F.D.); yn2469836834@163.com (N.Y.); zhang1692022@163.com (C.Z.); wangzhiwen981112@163.com (Z.W.); tangjiawei1020@163.com (J.T.); 2 White Lotus Industrial Development Center of Guangchang County, Fuzhou 344900, China; xwzheng@126.com (X.Z.); yangliangb@126.com (L.Y.)

**Keywords:** metabolite, sacred lotus, transcriptome, terpenoids, transcription factors

## Abstract

As the largest group of structurally diverse metabolites, terpenoids are versatile natural compounds that act as metabolism mediators, plant volatiles, and ecological communicators. However, few terpenoid compounds have been identified in plant parts of sacred lotus (*Nelumbo nucifera* Gaertn.). To elucidate the molecular genetic basis of the terpene biosynthetic pathway, terpenes from different parts of the plant, including seeds (S), young leaves (YL), mature leaves (ML), white flowers (WF), yellow flowers (YF), and red flowers (RF), were identified by LC-MS/MS and the relative contents of the same terpenes in different parts were compared. The results indicate that all plant parts primarily consist of triterpenes, with only minor quantities of sesquiterpenes and diterpenes, and there were differences in the terpene content detected in different plant parts. To illustrate the biosynthesis of various terpenoids, RNA sequencing was performed to profile the transcriptomes of various plant parts, which generated a total of 126.95 GB clean data and assembled into 29,630 unigenes. Among these unigenes, 105 candidate unigenes are involved in the mevalonate (MVA) pathway, methyl-erythritol phosphate (MEP) pathway, terpenoid backbone biosynthesis pathway, and terpenoid synthases pathway. Moreover, the co-expression network between terpene synthase (TPS) and WRKY transcription factors provides new information for the terpene biosynthesis pathway.

## 1. Introduction

Sacred lotus belongs to Nelumbonaceae, which contains only two species, sacred lotus (*Nelumbo nucifera* Gaertn.) and American lotus (*Nelumbo lutea* Pers.) [1]. Sacred lotus is one of the economically best-known aquatic plants in East Asia, with a history of cultivation for nearly 2000 years in China, and is commonly used for medicine and ornamentation [2,3]. A large number of metabolites, including alkaloids, steroids, flavonoids, triterpenoids, glycosides, and polyphenolshave, were widely detected in sacred lotus and are tightly related to their pharmacological activities, such as anti-ischaemia, antioxidant, anticancer, antiviral, as well as anti-obesity [4,5,6,7,8,9].

As the largest group of structurally diverse metabolites, terpenoids are versatile natural compounds that act as metabolism mediators, plant volatiles, and ecological communicators. Triterpenoids are important plant secondary metabolites with pharmacological effects, such as anticancer, antiviral, and cholesterol lowering. According to the number of rings in the structure, triterpenoids can be classified into monocyclic triterpenes, bicyclic triterpenes, tricyclic triterpenes, tetracyclic triterpenes, and pentacyclic triterpenes, etc. Among them, pentacyclic triterpenoids have received more attention because of more types and functions [10,11,12,13]. Biologically, triterpene saponins are considered to be defensive compounds against external stresses [14,15]. Terpenoids are sequentially biosynthesized from the universal C5 precursors as dimethylallyl diphosphate (DMAPP) and isopentenyl diphosphate (IPP) [16,17,18]. These C5 precursors are sequentially catalyzed by prenyltransferases to form prenyl diphosphates, including geranyl pyrophosphate (GPP), farnesyl pyrophosphate (FPP), and geranylgeranyl pyrophosphate (GGPP). These prenyl diphosphates are further used as the immediate precursors for the biosynthesis of various terpenoids, such as monoterpenes (C10), sesquiterpenes (C15), diterpenes (C20), triterpenes (C30), tetraterpenes (C40), as well as polyterpenes (more than C40) by the action of terpene synthase (TPS). Transcription factors (TFs) regulate the accumulation of terpenoids by activating or repressing the promoters of TPS genes to control their expression and five TFs have been identified as being involved in the regulation of terpene synthesis [19,20].

Despite the scientific and industrial interest in sacred lotus, terpenoid components have not been identified. Moreover, the genes involved in terpenoid biosynthesis were been completely studied in aquatic botany. In recent years, RNA sequencing (RNA-Seq) and metabolite analysis were developed as powerful tools for identifying genes involved in the biosynthesis of various secondary metabolites in higher plants, including *Salvia officinalis* Linn. [21], *Cinnamomum camphora* (L.) Presl. [10], and *Salvia guaranitica* St. Hil. [22].

As one of the best-known medicinal plants, few terpenoid compounds have been identified in plant parts of *N. nucifera*, and the molecular genetic basis of terpenoid biosynthesis pathways is still unveiled. Considering the specific characteristics of sacred lotus, it was of interest to profile the terpenoid biosynthesis and key genes related with the terpenoid metabolic pathway. In this study, the contents and compositions of terpenoids in seeds, young leaves, mature leaves, and flowers with different colors were analyzed with LC-MS/MS. In addition, the key genes and transcription factors involved in terpenoid biosynthesis were screened by RNA-seq. We believe these results will shed light on the mechanism of terpenoid biosynthesis in sacred lotus.

## 2. Results

### 2.1. Identification of Terpenoid Components in Various Plant Parts

**Figure 1:**
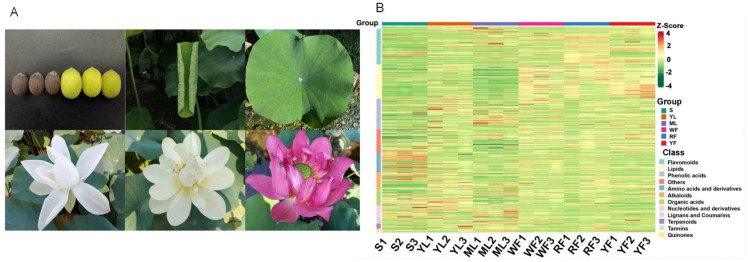
Metabolic analysis of various plant parts in sacred lotus. (**A**) Sample morphology of various plant parts. (**B**) Sample cluster map. The horizontal is the sample name, the vertical is the metabolite information, and the different colors are the values obtained after the relative content standardization process.

For unveiling the distribution of terpenoids in flowers with different colors, the widely planted cultivars with a red flower (Jinlinghuodu), white flower (Baiyinlian), and yellow flower (Jinsenianhua) were selected. In order to compare terpenoids in various plant parts, the seeds, young leaves, and mature leaves from Taikonglian 36 were harvested (Figure 1A). In total, 909 metabolites were detected in six sets of samples (three biological replicates per set) of lotus based on the UPLC-MS/MS detection platform and the self-constructed database. Because a broad-target metabolomic analysis was performed, which had some limitations for detecting each class of triterpenoids, cluster analysis of metabolites revealed that the 909 metabolites could be classified into 12 classes (Figure 1B). In addition, a total of 21 terpenoids were identified in different plant parts, and among these 21 terpenoids, 16 terpenoids could be detected in all plant parts, but their contents appeared different in these plant parts (Table 1). 

### 2.2. RNA-Seq and Transcriptomic Assembly

To identify genes involved in terpenoid biosynthesis in sacred lotus, 18 RNA libraries were prepared and analyzed. In total, 249,600,000 reads were obtained from these 18 libraries (accession number: PRJNA857167). After filtering the original data, clean reads were obtained for subsequent analysis (Table S1). Additionally, clean reads were mapped to the sacred lotus genome; the percentage of sequencing reads generated by each sample successfully aligned to the genome was higher than 80%. These results indicated that the quality of these mapped genes was sufficient to conduct the subsequent analysis (Table S2).

### 2.3. Gene Annotation and Functional Classification

**Figure 2:**
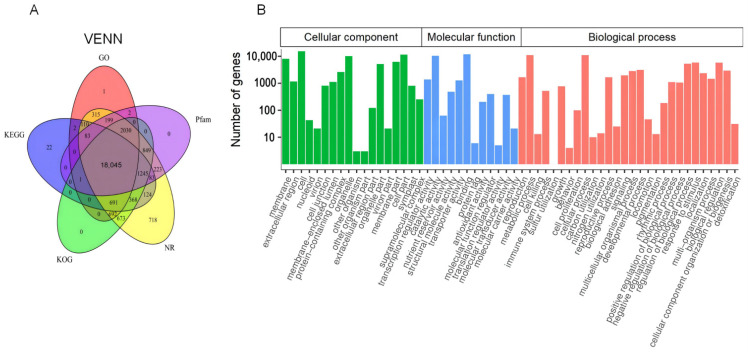
Annotation information of assembled unigenes in sacred lotus. (**A**) Venn diagram of the distribution of annotation information from different public databases. (**B**) GO classification of unigenes. The abscissa represents the secondary GO entry and the ordinate represents the number of genes in the GO entry. The secondary GO entry includes three parts: molecular function, biological process, and cell composition.

BLAST alignment was utilized to annotate the 29,630 unigenes of sacred lotus with an E-value threshold of 1e^−5^ in the public databases: NR (NCBI non-redundant protein sequences), KOG (euKaryotic Ortholog Groups), PFAM (Protein family), GO (Gene Ontology), and KEGG (Kyoto Encyclopedia of Genes and Genomes). In summary, 76,070 unigenes were successfully annotated by at least one database and 18,045 unigenes shared annotation in all databases (Figure 2A).

Based on sequence homology, 22,171 annotated unigenes were categorized into three ontologies with 58 GO terms (Figure 2B). Within the category of cellular component (CC), genes matched to 18 GO terms, the most highly represented of which were ‘cell’ (15,881 unigenes), ‘cell part’ (15,817 unigenes), and ‘cellular process’ (13,402 unigenes). For the molecular function (MF) category, ‘binding’ (12,464 unigenes) and ‘catalytic activity’ (10,985 unigenes) were the two most abundant of 12 GO terms. The largest associated term within the 28 GO terms of the biological process (BP) was ‘cellular process’ (13,402 unigenes).

In total, 25,512 unigenes were matched in 134 metabolic pathways and the biosynthesis of secondary metabolites (3964 unigenes), metabolic pathways (2194 unigenes), and plant–pathogen interaction (534) were the three richest pathways. In addition, 122 unigenes were mapped to the ‘Metabolism of terpenoids’, including ‘Terpenoid backbone biosynthesis’ (ko00900, 64 unigenes), ‘Sesquiterpenoid and triterpenoid biosynthesis’ (ko00909, 25 unigenes), and ‘Diterpenoid biosynthesis’ (ko00904, 33 unigenes) (Figure S1).

### 2.4. Identification of Differential Expression Genes (DEGs) 

**Figure 3:**
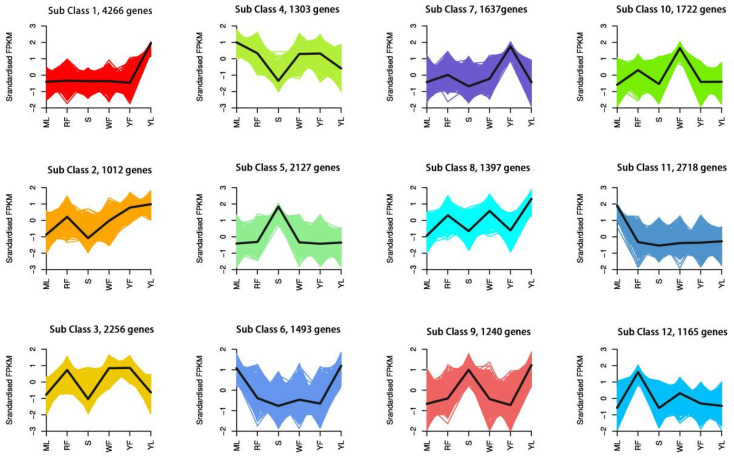
Clusters of DEGs. Twelve clusters were classified based on gene expression pattern: Sub Class1–Sub Class12. The horizontal coordinate represents the sample and the vertical coordinate represents the centralized and normalized expressions.

To fully explore potential DEGs in various plant parts, 22,336 DEGs were identified by comparing the eleven groups (S vs. ML, S vs. RF, S vs. WF, S vs. YF, S vs. YL, WF vs. RF, WF vs. YF, YL vs. ML, YL vs. WF, and YL vs. RF). The total number of DEGs, up-regulated DEGs, and down-regulated DEGs in each group are presented in Table S3. To further characterize the expression patterns of differential genes, we performed k-means clustering analysis on all DEGs. The results showed that 22,336 DEGs were assigned to 12 clusters with different numbers of DEGs per cluster, ranging from 1012 to 4266 (Figure 3). 

### 2.5. Functional Classification of DEGs

**Figure 4:**
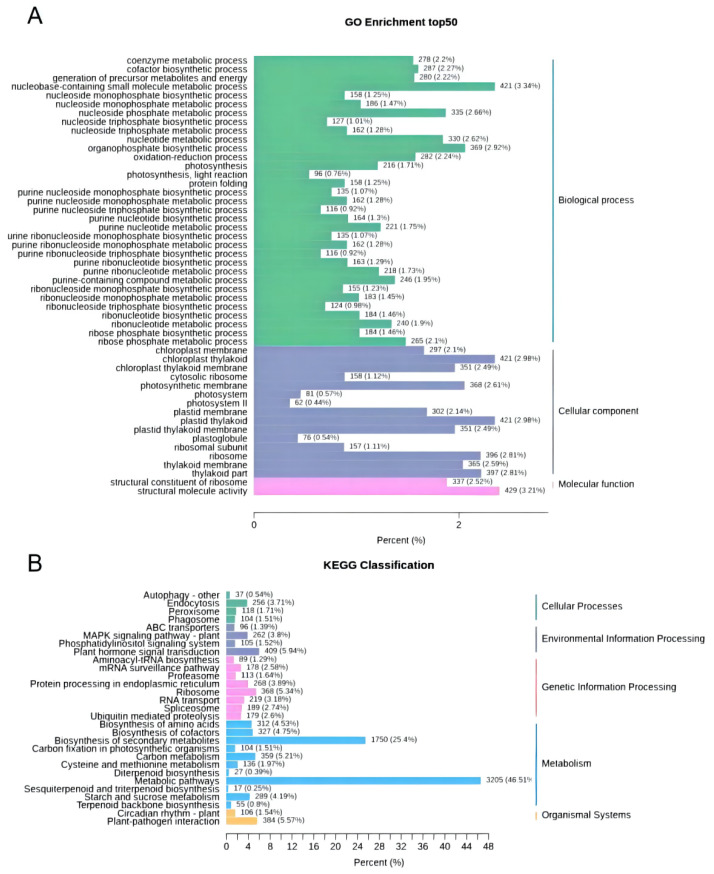
Annotation information of DEGs. (**A**) GO enrichment histogram of DEGs. The abscissa indicates the ratio of genes annotated to the entry to the total number of genes and the ordinate indicates the name of the GO entry. All DEGs were divided into three GO entries: biological process, cellular component, and molecular function. (**B**) KEGG classification column chart. DEGs were divided into five branches according to the KEGG metabolic pathway: Cellular Processes, Environmental Information Processing, Genetic Information Processing, Metabolism, and Organismal Systems.

The 50 GO-Terms with the lowest q-value in the enrichment analysis results were selected and a column chart of the enrichment items was drawn (Figure 4A). Among the 33 GO terms of biological process (BP), the largest related term was “cell process”. For the Cellular component (CC) category, the DEGs mainly participated in the chloroplast thylakoid, plastid thylakoid, and other organelles. In the category of molecular function (MF), “structural molecule activity” was the largest related term.

According to the KEGG pathway enrichment analysis, biosynthesis of secondary metabolites (1750) and metabolic pathways (3205) were the two richest pathways. In addition, 99 DEGs were positioned as “terpenoid metabolism”, including “Terpenoid backbone biosynthesis” (ko00900, 55), “sesquiterpene and triterpene biosynthesis” (ko00909, 17), and “diterpene biosynthesis” (ko00904, 27) (Figure 4B).

### 2.6. DEGs Involved in Terpenoid biosynthesis

**Figure 5:**
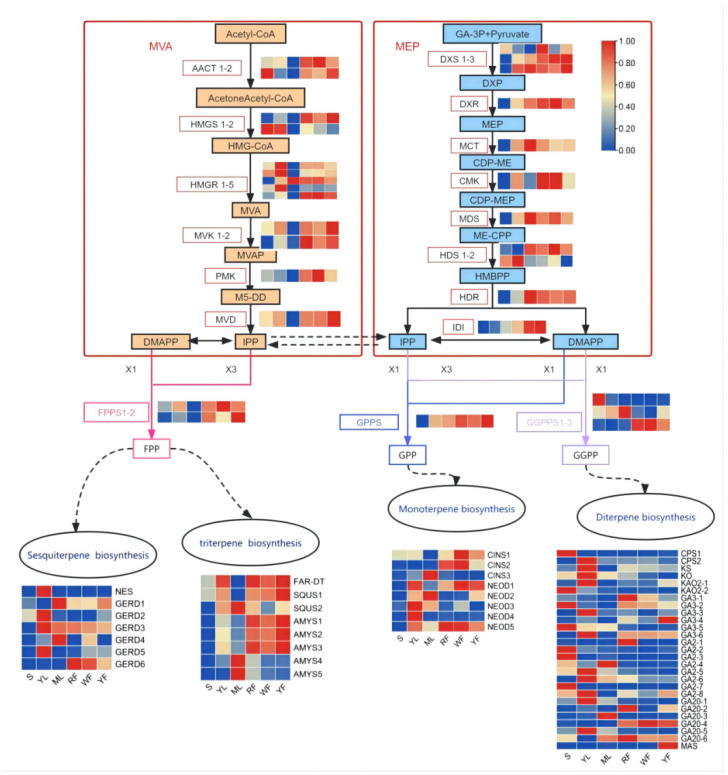
DEGs in terpenoid biosynthesis of sacred lotus. Typical terpenoid biosynthetic pathway with homology heat map of gene transcription level in transcriptome data with substrates and products. The arrows connect the substrate to their corresponding product. The expression pattern of each unigene is displayed in a six-column grid; from left to right are seed, young leaf, mature leaf, red flower, white flower, and yellow flower. Transcript-level data is represented by FPKM. *AACT*, *Acetyl-CoA C-acetyltransferase; HMGS, hydroxymethylglutaryl-CoA synthase; HMGR, hydroxymethylglutaryl-CoA reductase (NADPH); MVK, mevalonate kinase; PMK, phosphomevalonate kinase; MVD, diphosphomevalonate decarboxylase; DXS, 1-deoxy-D-xylulose-5-phosphate synthase; DXR, 1-deoxy-D-xylulose-5-phosphate reductoisomerase; MCT, 2-C-methyl-D-erythritol 4-phosphate cytidylyltransferase; CMK, 4-diphosphocytidyl-2-C-methyl-D-erythritol kinase; MDS, 2-C-methyl-D- erythritol 2,4-cyclodiphosphate synthase; HDS, (E)-4-hydroxy-3-methylbut-2- enyl-diphosphatesynthase; HDR, 4-hydroxy-3-methylbut-2-en-1-yl diphosphate reductase; IDI, isopentenyl-diphosphate Delta-isomerase; DMAPP, dimethylallyl diphosphate; GPPS, geranyl diphosphate synthase; FPPS, farnesyl diphosphate synthase; GGPPS, geranylgeranyl diphosphate synthase; NRS, (3S,6E)-nerolidol synthase; GERD, (-)-germacrene D synthase; FAR-DT, farnesyl-diphosphate farnesyltransferase; SQUS, squalene monooxygenase; AMYS, beta-amyrin synthase; CINS, 1,8-cineole synthase; NEOD, (+)-neomenthol dehydrogenase; CPS, ent-copalyl diphosphate synthase; KS, ent-kaurene synthase; KO, ent-kaurene oxidase; KAO2, ent-kaurenoic acid oxidase 2; GA3, gibberellin 3-beta-dioxygenase; GA2, gibberellin 2-oxidase; GA20, gibberellin 20-oxidase; MAS, momilactone-A synthase*.

To unveil the regulatory mechanism of terpenoid accumulation patterns in different plant parts of sacred lotus, we analyzed the expression profiles of genes involved in terpenoid biosynthesis. In total, 105 DEGs related to terpenoid biosynthesis were identified in sacred lotus and their expression patterns are shown in Figure 5. In total, 55 DEGs were annotated to the terpenoid backbone synthesis pathway (ko00900), of which 13 and 11 DEGs participated in the MVA and MEP pathways, respectively. Furthermore, DEGs encoding key enzymes in the MVA pathways exhibited a higher expression level in YL compared with S and ML. Interestingly, *AACT2* and *HMGS2* exhibited the highest mRNA level in S, while *HMGR3* was the highest expressed in ML (Figure 5 and Table S4). Similar to the MVA pathway, the expression levels of DEGs involved in MEP in leaves was still higher than seeds, and the expression levels of DEGs in ML were significantly higher than YL (Figure 5 and Table S4). Moreover, six unigenes encoding IPTS were identified, including geranyl diphosphate synthase (GPPS), farnesyl diphosphate synthase (FPPS), and geranylgeranyl diphosphate synthase (GGPPS). The expression level of *FPPS* in YL was higher than that in S and ML and also had higher transcript levels in the three differently colored flowers, which was consistent with the expression pattern of DEGs in the MVA pathway. The expression level of *GPPS* in S was lower than in other parts, but there was no obvious difference in leaves and flowers. The different expression patterns in the three *GGPPS* transcripts are worth noting. The *GGPPS2* transcript exhibited the highest expression level in mature leaves, with the highest mRNA level of *GGPPS3* in WF, RF, and YF. Moreover, the expression level of *GGPPS1* was relatively lower than that of *GGPPS2* and *GGPPS3* (Figure 5 and Table S4). 

**Figure 6:**
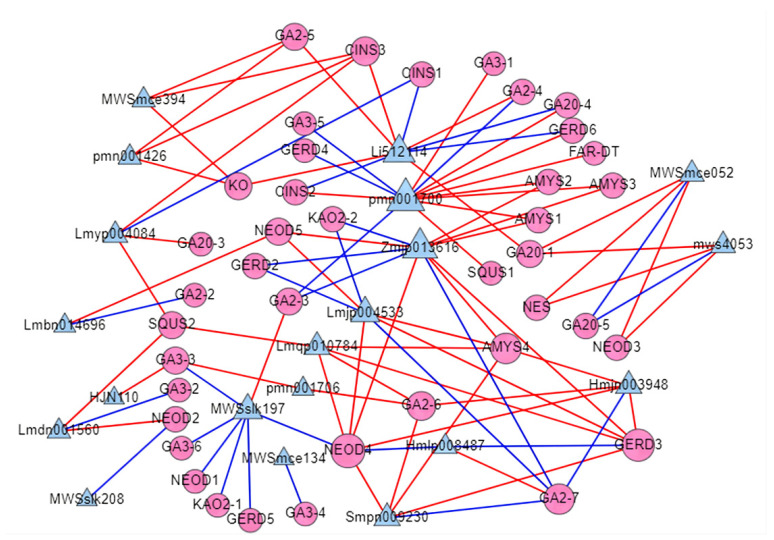
Correlation analysis of TPS genes and released amounts of terpene compounds (Pearson correlation coefficient > 0.8). The circular nodes are TPS genes, the triangular nodes are terpenes, the red line represents positive correlation, and the blue line represents negative correlation.

As key enzymes in the synthesis of terpenoids, 49 DEGs encoding terpenoid synthase (TPS) were found, including 15 genes involved in sesquiterpene and triterpene synthesis (ko00909), 26 genes involved in diterpene synthesis (ko00904), and 8 genes involved in monoterpene synthesis (K07385 and K15095) (Table S5). Correlation network analysis was used to model the relationship of the selected 49 genes encoding TPS with the detected terpenoids (Figure 6). As shown in the figure, 40 TPS genes and 20 terpenoids showed high correlation, for example, (-)-germacrene D synthase (*GERD3*) was positively correlated with five terpenoids, including Triacetate (lmjp04533), 12,13-Dihydroursolic acid (Zmjp01361), Progesterone (Lmqp010784), 2α,3α,23-trihydroxyolean-12-en-28-oic acid (Smpn009230), and Madasiatic acid (Hmjn003948), with correlation coefficients varying from 0.83 to 0.97. Gibberellin 2 beta-dioxygenase (GA2-5), 1,8-cineole synthase (CINS3), and ent-kaurene oxidase (KO) were positively correlated with the same three terpenoids. 24,30-Dihydroxy-12(13)-enolupinol (pmn001700) was positively correlated with nine TPS genes and negatively correlated with four TPS genes. Beta-amyrin synthase (AMYS1, AMYS2, AMYS3) exhibited a strong positive correlation with 24,30-Dihydroxy-12(13)-enolupinol (pmn001700) and 12,13-Dihydroursolic acid (Zmjp013616).

### 2.7. Co-Expression Network of TPS and WRKY Transcription Factors

**Figure 7:**
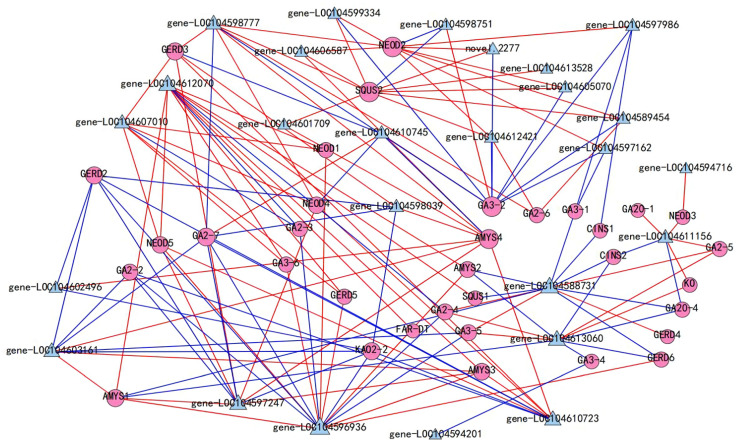
Correlation analysis of TPS genes and WRKY transcription factors (Pearson correlation coefficient > 0.8). The circular nodes are TPS genes, the triangular nodes are WRKY, the red line represents positive correlation, and the blue line represents negative correlation.

Transcription factors (TFs) usually control the expression of TPS genes by activating or repressing their promoters, thereby regulating the accumulation of terpenoids. In total, 47 DEGs were annotated as WRKY transcription factors, 27 of which were highly correlated with 15 terpenoids (Table S6, Figure S2). A co-expression network of these 27 WRKY transcription factors and the 40 genes encoding TPS was constructed (Figure 7). The results showed that WRKY9 (gene-LOC104588731) was negatively correlated with *GERD2*, *GA2-2*, *GA2-7*, and *KAO2-2*, which was positively correlated with *GERD3*, (+)-neomenthol dehydrogenase (*NEOD4, NEOD5*), *AMYS3*, and *AMYS4*. WRKY72 (LOC104596936) was closely related to 15 *TPS* genes. Among them, WRKY72 was positively correlated with *NEOD5*, *AMYS1*, *AMYS2*, and *AMYS3*, and the Pearson correlation coefficient exceeded 0.9. By contrast, WRKY72 was negatively correlated with *GA2-2*, *GA2-3*, *GA2-4*, *GA2-7*, gibberellin 3-beta-dioxygenase (*GA3-5*), GERD2, and ent-kaurenoic acid hydroxylase (*KAO2-2*). WRKY17 (LOC104603161) was positively correlated with *AMYS1*, *AMYS3*, and *AMYS4*. 

### 2.8. qRT-PCR Validation of DEGs

**Figure 8:**
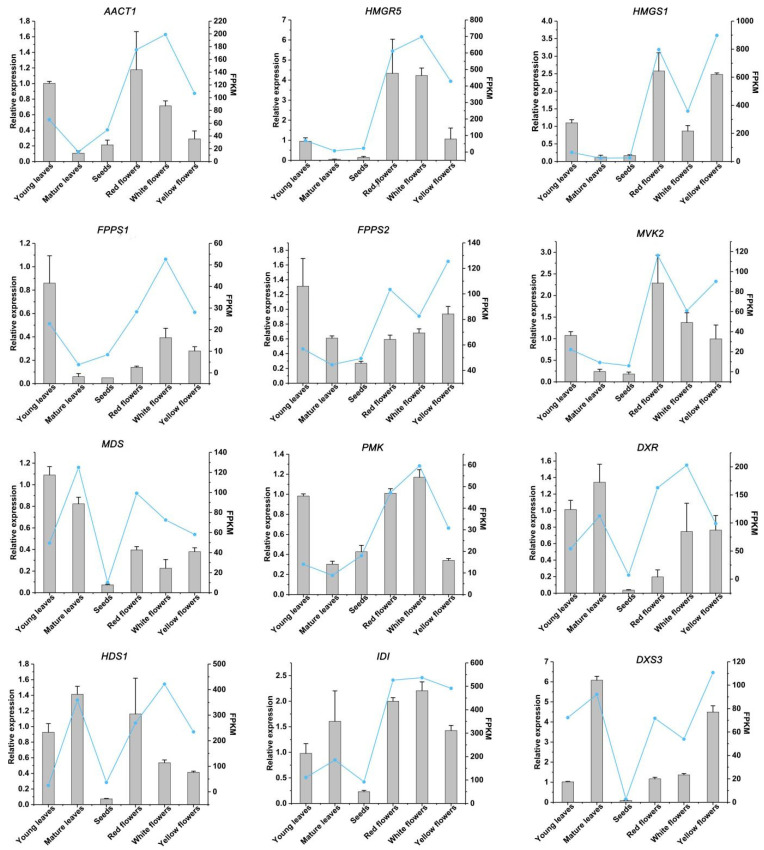
qRT-PCR and RNA-Seq analysis of 12 terpenoid-pathway-related candidate unigenes in sacred lotus. FPKM terms of various plants parts were determined by RNA-seq. Relative expression of genes was calculated using young leaves as control. Standard error of the mean for three biological replicates is represented by the error bars. *AACT*, Acetyl-CoA C-acetyltransferase; *HMGS*, *hydroxymethylglutaryl-CoA synthase*; *HMGR*, *hydroxymethylglutaryl-CoA reductase (NADPH)*; *FPPS*, *farnesyl diphosphate synthase*; *MVK*, *mevalonate kinase*; *MDS*, *2-C-methyl-D- erythritol 2,4-cyclodiphosphate synthase*; *PMK*, *phosphomevalonate kinase*; *DXR*, *1-deoxy-D-xylulose-5-phosphate reductoisomerase*; *IDI*, *isopentenyl-diphosphate Delta-isomerase*; *DXS*, *1-deoxy-D-xylulose-5-phosphate synthase*.

To verify the reliability of the RNA-Seq data, the expression of 12 unigenes was detected by qRT-PCR (Figure 8). The expression levels of these genes determined by qRT-PCR were almost consistent with those inferred from the RNA-Seq FPKM data (Figure 8), except for the expression pattern of *AACT1* and *FPPS1* in white flower.

## 3. Discussion

The remarkable health and disease alleviation activities of the scared lotus are associated with the high content of bioactive compounds, including polyphenols, flavonoids, phenolic acids, alkaloids, terpenoids, steroids, fatty acids, and glycosides [23]. However, most studies focus on flavonoids, alkaloids, and phenolic acids, while few have been conducted on terpenoids [24,25,26]. In this study, in total, 21 terpenoids were detected (Table 1). Although the genes involved in terpenoid biosynthesis are widely studied in different plants [20,27,28,29,30,31,32,33], genes related to the synthesis of terpenoids in sacred lotus have not been studied so far. The precursors of monoterpenes and diterpenes come from the MEP pathway in plastids, while the precursors of sesquiterpenes and triterpenes come from the MVA pathway in the cytoplasm [34]. Considering the two rate-limiting enzymes, HMGR and DXS played important roles in the overall regulation of the MVA and MEP pathways, respectively [35,36,37]. Among five genes encoding HMGR identified in this study, *HMGR1*, *HMGR2*, and *HMGR4* exhibited the highest expression in YL, with the lowest expression in ML (Figure 5). In total, three genes encoding DXS were detected; *DXS2* and *DXS3* had the same expression pattern, with higher expression in leaves than in seeds, and there was no significant difference in expression in the three colors of flowers, which corresponded to the metabolic content results. This result indicated that the distribution of terpenoids in different plant parts was closely related to the expression pattern of genes involved in the MVA and MEP pathways. However, the relative contribution of each pathway to the biosynthesis of various terpenoids was still uncertain.

IPTS are the key enzymes that connect the upstream MVA and MEP pathways with downstream isoprenoid biosynthesis branch points of different structures. In recent years, GPPS, GGPPS, FPPS, and CPPS, as the key enzymes in the biosynthesis of isoprenoid compounds, were identified in higher plants. In addition, most studies showed that the transcription levels of these genes were closely related to the content of corresponding terpenoids [38,39,40,41]. For example, FPPS overexpression increased the production of ganoderic acid [42]. A high number of monoterpenes was produced by overexpression of peppermint (*Mentha × piperita*) *GPPS.SSU* in transgenic tobacco plants [43] and the content of abietane diterpenes in *Salvia sclarea* L. hairy roots was increased by engineering the *GGPPS* and *CPPS* [44]. In this research, six DEGs encoding IPTS were identified, including one *GPPS*, two *FPPS*, and three *GGPPS*. We found that the expression of *FPPS* was higher in young leaves than in seeds and mature leaves, which is consistent with the results detected for the metabolic content of triterpenoids. It has been shown that the *GGPPS* transcript was mainly concentrated in the above-ground parts, such as leaves, flowers, or fruits, and the expression patterns of different *GGPPS* homologous genes in the same plant were various [45,46,47,48,49,50,51]; the expression patterns of *GGPPS2* and *GGPPS3* identified in this study are consistent with this conclusion.

As a key enzyme playing an important role in the synthesis of terpenoids, the TPS genes were identified in *A. thaliana*, tomato, rice, and tobacco [52,53,54,55,56]. Further, a large number of different TPS and their products were the chief reasons for the variety in terpenes [57,58]. In this study, 40 DEGs encoding TPS exhibited a high correlation with 20 terpenes (Figure 6). In previous studies, multiple TPS in most plants were shown to be multi-product enzymes [58]. Our study indicated that most TPS in lotus also showed a high correlation with multiple products and some terpenoids were also highly correlated with multiple TPS. Therefore, it is speculated that these terpenoids may be regulated by multiple TPS. WRKY transcription factors have been reported to regulate terpenoid production by activating or inhibiting promoter binding of TPS [20,59,60,61]. In our study, our results showed that 26 members of WRKY were closely related to 34 TPS genes (Figure 7). These phenomena suggest that WRKY transcription factors play an important role in the regulation of terpenoid production, but the exact mode of action needs to be further explored and confirmed.

## 4. Materials and Methods

### 4.1. Plants Materials

The widely planted cultivars “Taikonglian 36”, “Jinsenianhua”, “Jinlinghuodu”, and “Baiyinlian” were planted in pools at Henan University of Technology, with a photoperiod of light for 16 h and dark for 8 h. Sacred loti were cultivated to the full-bloom stage. The materials including seeds (Taikonglian 36), young leaves (Taikonglian 36), mature leaves (Taikonglian 36), and blooming flowers with red (Jinlinghuodu), white (Baiyinlian), and yellow (Jinsenianhua) were freeze dried in liquid nitrogen and kept at −80 °C.

### 4.2. LC-MS/MS Analysis

Biological samples were freeze dried using a vacuum freeze dryer (Scientz-100F) and crushed using a mixer mill (MM 400, Retsch) with a zirconia bead for 1.5 min at 30 Hz. Then 100 mg of lyophilized powder was dissolved with 1.2 mL 70% methanol solution, and vortexed 30 s every 30 min 6 times in total. The samples were refrigerated 4 °C overnight and centrifugated at 12,000 rpm for 10 min. The extracts were filtrated (SCAA-104, 0.22 μm pore size; ANPEL, Shanghai, China, http://www.anpel.com.cn/, accessed on 5 September 2021) before UPLC-MS/MS analysis. Chromatography and mass spectrometry were performed with the assistance of Wuhan Metware Biotechnology Co., Ltd. (Wuhan, China) as the method described [62]. Metabolite quantification was accomplished by multiple reaction monitoring (MRM) analysis using triple-quadrupole mass spectrometry. The characteristic ions of each substance were screened by triple quadruple rods, the signal intensity (CPS) of the characteristic ions was obtained in the detector. The sample offline mass spectrometry file was opened with MultiaQuant software. The integration and calibration of the peaks were performed, and the peak area (Area) of each peak represented the relative content of the corresponding substance. The internal standard used was 2-chloro-L-phenylalanine (CAS: 103616-89-3, Bailiwick, 106151-100 mg). The HCA (hierarchical cluster analysis) results of metabolites were presented as heatmaps and normalized signal intensities of metabolites (unit variance scaling) were visualized as a color spectrum.

### 4.3. Total RNA Extraction and Transcriptome Sequencing

Total RNA was extracted using the Total Plant RNA Extraction Kit (Tsingke, Beijing, China). RNA concentration and purity were determined using NanoPhotometer^®^ spectrophotometer (IMPLEN, München, BY, GER) and RNA concentration was measured using Qubit^®^ RNA Assay Kit in Qubit^®^2.0 Flurometer (Life Technologies, Waltham, CA, USA). RNA integrity was assessed using the RNA Nano 6000 Assay Kit of the Bioanalyzer 2100 system (Agilent Technologies, Palo Alto, CA, USA). A total amount of 1 µg RNA per sample was used as input material for the RNA sample preparations. Sequencing libraries were generated using NEBNext^®^ UltraTM RNA Library Prep Kit for Illumina^®^ (NEB, Ipswich, MA, USA) following manufacturer’s recommendations. The cDNA libraries were sequenced on the Illumina sequencing platform by Metware Biotechnology Co., Ltd. (Wuhan, China).

### 4.4. RNA-Seq Analysis

The original data were filtered to remove reads with adapters. HISAT version 2.1.0 (Daehwan Kim, Baltimore, MA, USA) was used to construct the index and compare clean reads to the reference genome (https://www.ncbi.nlm.nih.gov/genome/?term=AQOG01, accessed on 25 September 2021) [63], and StringTie version 1.3.4d (Mihaela Pertea, Baltimore, MA, USA) was used for new gene prediction [64]. We used featureCounts version 1.6.2 (Yang Liao, Parkville, VIC, AUS) to calculate the gene alignment and then calculated the transcript per million fragments mapped (FPKM) of each gene based on the gene length. DESeq2 version 1.22.1 (Michael I Love, Heidelberg, BW, GER) [65,66] was used to analyze the original count data and to screen for DEGs, and genes satisfying |log2Fold Change| ≥ 1 and False Discovery Rate (FDR) < 0.05 were defined as differentially expressed genes (DEGs). The enrichment analysis was performed based on the hypergeometric test. The gene co-expression network analysis was performed using the Metware Cloud, a free online platform for data analysis (https://cloud.metware.cn, accessed on 3 January 2022), and Pearson correlation coefficient greater than |0.8|.

### 4.5. Validation of DEGs by qRT-PCR Analysis

The key DEGs in the terpenoid synthesis pathway were selected to validate their expression levels by quantitative reverse transcription-polymerase chain reaction (qRT-PCR). Synthesis of cDNA for qRT-PCR was performed with First Strand cDNA Synthesis Kit (ThermoFisher Scientific, Shanghai, China). The primers were synthesized by Tsingke Biotechnology Co., Ltd. (Wuhan, China) (Table S7). The reaction system of qRT-PCR was as follows: cDNA template 0.5 μL at 3000 ng/μL, gene-specific upstream and downstream primers 0.4 μL each at 10 μM, 2 × ChamQ Universal SYBR qPCR Master Mix (Vazyme, Nanjing, China) 10 μL, 8.7 μL of double-distilled water (ddH2O). qRT-PCR was performed by CFX96 Real Time System (Bio-Rad, Hercules, CA, USA). Young leaves were selected as the control and 26s rRNA as the internal reference genes. The relative expression of genes in the qRT-PCR experiment was analyzed by the 2^−^^△△CT^ method [67]. At least three biological replicates were set for each sample.

## 5. Conclusions

In this study, 21 terpenoids were detected in sacred lotus and 105 genes involved in terpenoid synthesis were identified. WRKY, an important transcription factor, is highly correlated with the TPS gene family and might play an important regulatory role in the terpenoid synthesis process. This study illustrated the metabolic diversity of terpenoids in sacred lotus and provided a global overview of the gene expression profiles related with terpenoid biosynthesis in sacred lotus.

## Figures and Tables

**Table 1:** Relative content of terpenoids.

Index	Compounds	Class II	Q1 (Da)	Q3 (Da)	Level	S	YL	ML	WF	RF	YF
Lmyp004084	Perillyl alcohol	Monoterpenoids	1.53 × 10^2^	1.07 × 10^2^	3	3.77 × 10^4^	7.46 × 10^4^	2.75 × 10^5^	1.42 × 10^4^	1.28 × 10^5^	3.54 × 10^4^
Hmlp008487	Blumenol C	Monoterpenoids	2.11 × 10^2^	8.11 × 10^1^	2	5.35 × 10^4^	3.68 × 10^4^	4.29 × 10^4^	4.13 × 10^4^	4.54 × 10^4^	4.00 × 10^4^
MWSslk208	Kaurenoic Acid	Ditepenoids	3.01 × 10^2^	3.01 × 10^2^	2	1.39 × 10^5^	3.99 × 10^4^	3.45 × 10^4^	1.92 × 10^5^	1.25 × 10^5^	2.12 × 10^4^
Lmbn014696	Pimaric acid	Ditepenoids	3.01 × 10^2^	3.01 × 10^2^	2	1.31 × 10^5^	4.26 × 10^5^	2.61 × 10^5^	6.73 × 10^5^	5.65 × 10^5^	2.27 × 10^5^
Lmqp010^7^84	Progesterone	Ditepenoids	3.15 × 10^2^	1.09 × 10^2^	2	4.26 × 10^4^	1.78 × 10^6^	1.10 × 10^6^	1.65 × 10^5^	2.05 × 10^6^	2.96 × 10^5^
Lmdn001560	6-DeoxyCatalpol	Sesquiterpenoids	3.45 × 10^2^	1.65 × 10^2^	2	7.79 × 10^4^	2.12 × 10^5^	3.96 × 10^5^	9.90 × 10^4^	1.53 × 10^5^	2.15 × 10^5^
Hmmn003964	7-Deoxyloganic acid	Sesquiterpenoids	3.59 × 10^2^	1.97 × 10^2^	3	1.17 × 10^5^	2.09 × 10^4^	4.41 × 10^4^	6.32 × 10^4^	4.45 × 10^4^	7.78 × 10^3^
MWSslk197	Secoxyloganin	Sesquiterpenoids	4.05 × 10^2^	1.65 × 10^2^	2	1.31 × 10^5^	9.96 × 10^3^	5.50 × 10^4^	3.76 × 10^4^	2.46 × 10^4^	2.01 × 10^4^
Lmjp004533	Kisasagenol A Triacetate	Ditepenoids	4.47 × 10^2^	2.85 × 10^2^	2	9.00 × 10^0^	6.08 × 10^4^	1.13 × 10^5^	3.12 × 10^4^	1.89 × 10^4^	6.32 × 10^4^
MWSmce134	Betulonic acid	Triterpene	4.53 × 10^2^	4.53 × 10^2^	3	4.16 × 10^3^	7.04 × 10^3^	3.15 × 10^3^	1.13 × 10^3^	9.60 × 10^2^	9.00 × 10^0^
pmn001700	24,30-Dihydroxy-12(13)-enolupinol	Triterpene	4.55 × 10^2^	4.55 × 10^2^	1	1.84 × 10^4^	5.13 × 10^4^	1.33 × 10^4^	3.60 × 10^5^	4.79 × 10^5^	3.42 × 10^5^
HJN110	Betulinic acid	Triterpene	4.55 × 10^2^	4.55 × 10^2^	2	1.30 × 10^5^	4.16 × 10^5^	1.77 × 10^5^	5.79 × 10^4^	1.64 × 10^5^	1.84 × 10^5^
mws4053	Ursolic acid	Triterpene	4.55 × 10^2^	4.55 × 10^2^	3	5.60 × 10^5^	4.24 × 10^6^	1.08 × 10^6^	5.73 × 10^5^	2.41 × 10^5^	4.01 × 10^5^
MWSmce052	3-Epiursolic acid	Triterpene	4.55 × 10^2^	4.55 × 10^2^	3	5.39 × 10^5^	4.05 × 10^6^	1.14 × 10^6^	5.63 × 10^5^	2.45 × 10^5^	3.94 × 10^5^
Zmjp013616	12,13-Dihydroursolic acid	Triterpene	4.59 × 10^2^	4.23 × 10^2^	3	9.00 × 10^0^	3.98 × 10^4^	1.62 × 10^4^	1.49 × 10^4^	1.89 × 10^5^	1.15 × 10^5^
pmn001706	2-Hydroxyoleanolic acid	Triterpene	4.71 × 10^2^	4.71 × 10^2^	1	6.68 × 10^5^	1.94 × 10^7^	5.08 × 10^6^	1.10 × 10^6^	1.00 × 10^7^	1.41 × 10^6^
Li512114	Corosolic Acid Methyl Ester	Triterpene	4.85 × 10^2^	4.53 × 10^2^	3	3.10 × 10^3^	5.62 × 10^3^	5.89 × 10^3^	1.18 × 10^3^	2.29 × 10^3^	1.32 × 10^3^
MWSmce394	Tormentic acid	Triterpene	4.87 × 10^2^	4.69 × 10^2^	3	3.62 × 10^3^	3.18 × 10^4^	1.52 × 10^4^	6.23 × 10^2^	6.18 × 10^3^	9.00 × 10^0^
Hmjn003948	Madasiatic acid	Triterpene	4.87 × 10^2^	4.87 × 10^2^	2	3.49 × 10^4^	1.87 × 10^6^	5.72 × 10^5^	2.35 × 10^5^	2.15 × 10^6^	6.11 × 10^5^
pmn001426	Euscaphic acid	Triterpene	4.87 × 10^2^	4.69 × 10^2^	3	3.18 × 10^3^	3.23 × 10^4^	1.60 × 10^4^	3.93 × 10^2^	6.52 × 10^3^	9.00 × 10^0^
Smpn009230	2α,3α,23-trihydroxyolean-12-en-28-oic acid	Triterpene	4.87 × 10^2^	4.87 × 10^2^	2	3.34 × 10^4^	1.88 × 10^6^	5.09 × 10^5^	2.27 × 10^5^	2.01 × 10^6^	5.99 × 10^5^

Notes: Index, Metabolite number, Class II, secondary classification of substances. Q1, the molecular weight of the parent ion after the substance was added to the ion by the electrospray ion source, Q3, the characteristic fragment ion, level, substance identification level, “1”, sample substance secondary mass spectra, RT and database substance matching score of 0.7 or more; “2”, sample substance secondary mass spectra, RT and database substance matching score of 0.5–0.7, “3”, sample substance’s five detection parameters Q1, Q3, RT, DP, CE, and database substance check are consistent. The values in the table are the relative content of metabolites, without units, calculated by calculating the peak area formed in the detector by the characteristic ions of each substance. It is not the absolute content of the substance, but the detection conditions are consistent and can be used to compare the differences in the same substance in different samples.

## Data Availability

Raw sequencing data were deposited in the NCBI Bioproject database (accession number: PRJNA857167, https://www.ncbi.nlm.nih.gov/bioproject/PRJNA857167, accessed on 17 July 2022).

## Supplementary Materials

The following supporting information can be downloaded at: https://www.mdpi.com/article/10.3390/molecules27144599/s1, Figure S1: KEGG annotation of unigenes. The unigenes were divided into five branches according to the KEGG metabolic pathway: Cellular Processes, Environmental Information Processing, Genetic Information Processing, Metabolism, and Organismal Systems, Figure S2: Correlation analysis of WRKY TFs and released amounts of terpene compounds (Pearson correlation coefficient > 0.8). The circular nodes are WRKY TFs, the triangular nodes are terpenes, the red line represents positive correlation, and the blue line represents negative correlation, Table S1: Sequencing output statistics, Table S2: Comparison efficiency statistics, Table S3: DEGS of groups, Table S4: The fpkm value of genes involved in terpene backbone synthesis, Table S5: The fpkm value of genes involved in terpenoid synthesis, Table S6: The fpkm value of genes annotated as WRKY transcription factors, Table S7: Primers used in qPCR.

## Sample Availability

Samples are not available.
