# Supplementary figures and images for: Transcriptome Analyses Revealed the Key Metabolic Genes and Transcription Factors Involved in Terpenoid Biosynthesis in Sacred Lotus
Source: Molecules. 2022 Jul 19;27(14):4599. doi: 10.3390/molecules27144599 (PMC9320166; doi:10.3390/molecules27144599)

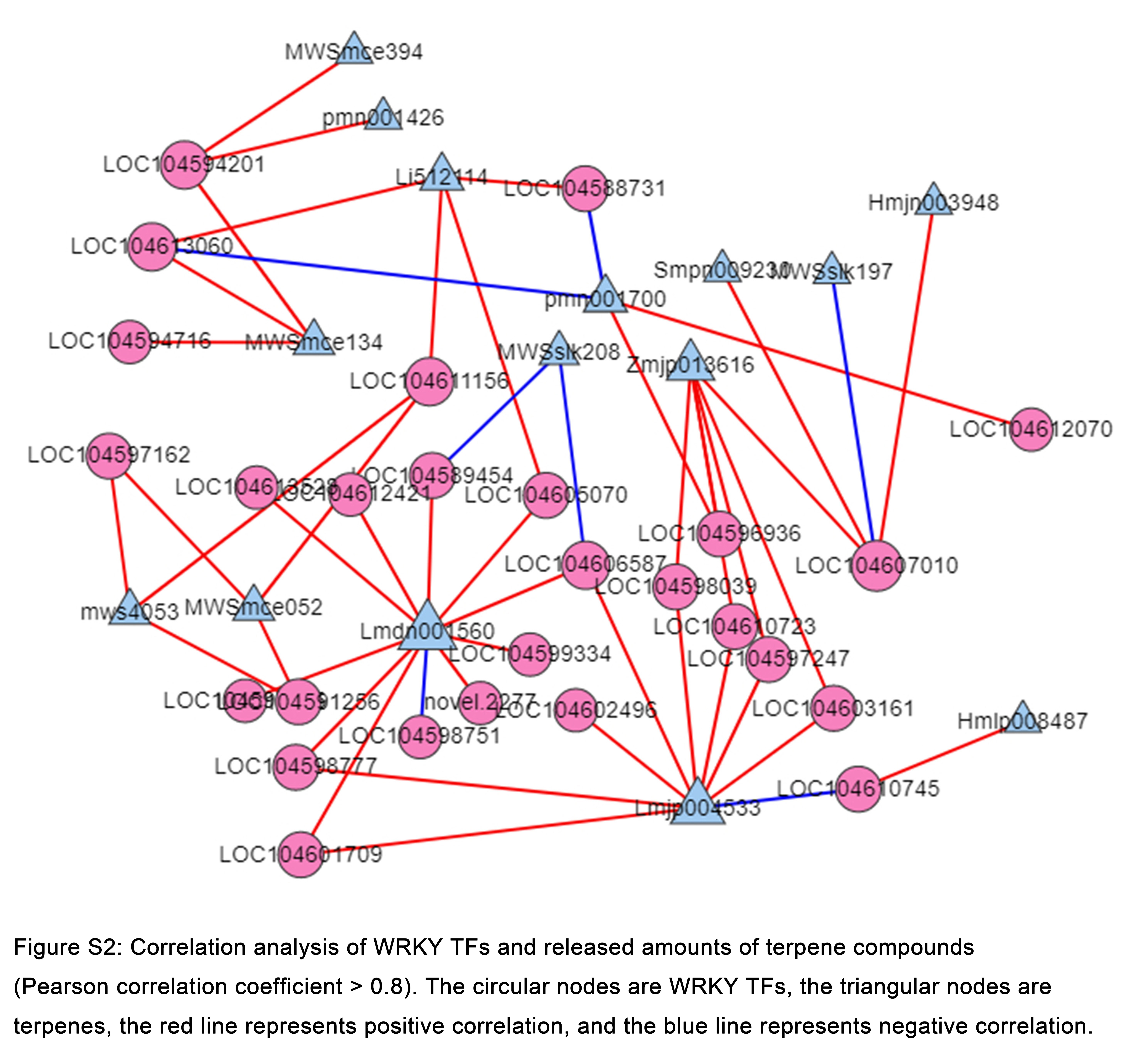

Supplement: Supplementary file 1 [file molecules-27-04599-s001.zip › Figure S2.jpg]
